# FAD-Linked Oxidoreductase Protein 1 (FLO1) Coordinates Grain Development and Drought Tolerance in Rice

**DOI:** 10.3390/plants15071100

**Published:** 2026-04-03

**Authors:** Uzair Ullah, Lubna Khan, Jia-Jun Ma, Zi Wang, Hong-Jin Wang, Munib Ahmad, Nadeem Bhanbhro, Yu-Xiang Huo, Abdullah Shalmani, Kun-Ming Chen

**Affiliations:** 1 National Key Laboratory of Crop Stress Resistance and High-Efficiency Production, College of Life Sciences, Northwest A&F University, Yangling 712100, China; 2 College of Biological Science and Engineering, Shaanxi University of Technology, Hanzhong 723000, China; 3 College of Life Science and Technology, Huazhong Agricultural University, Wuhan 430070, China

**Keywords:** rice (*Oryza sativa* L.), grain size, drought tolerance, FAD-linked oxidoreductase protein, OsWRKY53

## Abstract

Rice grain yield and drought tolerance are critical for global food security. So far, only a few genes have been reported to regulate both traits simultaneously. Here, we characterize OsFLO1, a previously unreported FAD-linked oxidoreductase, as a dual regulator of grain development and drought stress tolerance in rice. Genome-wide association studies (GWAS) revealed natural variation in *OsFLO1*, with haplotypes showing geographic adaptation to local rainfall. Functional analysis demonstrated that overexpression (OX) lines exhibited larger grains and improved panicle traits, while knockout (CR) lines showed reduced grain size and yield components despite increased tiller number. Regarding drought tolerance, OX lines of *OsFLO1* enhanced drought tolerance, as evidenced by increased root length and antioxidant activities, whereas knockout (CR) lines displayed impaired stress responses. We further show that OsWRKY53 directly binds the *OsFLO1* promoter, thereby activating its expression and coordinating both grain development and stress responses. Together, these results suggest that OsFLO1 functions as a key regulator coordinating grain development and drought tolerance, making it a promising target for improving rice productivity.

## 1. Introduction

Rice (*Oryza sativa* L.) is the primary food source for more than 50% of the world’s population, and the grain-related characteristics, including grain size, grain weight, grain-filling efficiency, and panicle architecture, mainly determine its productivity. Each of these characteristics is highly susceptible to environmental stressors, particularly drought, which occurs most frequently during the reproductive and grain-filling periods of rice production [1,2]. During these crucial growth periods, drought causes a breakdown in assimilate transport, disrupted hormone distribution, and alteration in the endosperm, all of which lead to reduced grain weight, decreased grain filling, and dramatic yield losses [3].

Grain size, shape, and quality in rice are regulated by a complex network of genes and signaling pathways that coordinate cell proliferation, elongation, and endosperm development. Regulatory modules such as OsEIL1-OsERF115 [4], OsSPL16-GW7 [5], and OsSPL4 [6], along with genes like *PsSPL13* [7], *OsPL18* [8], *SMALL GRAIN 11* [9], *GL10* [10], *GS3* [11], *qGL3* [11], and *GR5* [12], modulate grain size and yield by controlling gene expression during panicle and grain development. Transcription factors and microRNA-mediated regulators, including OsGRF4-OsmiR396 [13], OsNF-YX10 [14], and FLOUR ENDOSPERM 2 [15], influence grain width, endosperm formation, and starch quality, while *FERONIA*-like receptor genes [16], *SLG7* [17], and the GSK2-LARGE1/OML4 [18] pathway adjust cell proliferation and elongation to fine-tune grain morphology. Additionally, *RGA1* [19] impacts grain size, rice quality, and seed germination, highlighting the interaction of growth, yield, and seed viability. Collectively, these genes and regulatory pathways orchestrate the genetic and physiological mechanisms underlying grain development, offering valuable targets for improving rice yield and quality.

Drought tolerance in rice is also mediated by a complex network of genes and regulatory modules that integrate transcriptional, hormonal, and physiological responses. For instance, genes such as *OsAHL1* [20], *OsGRAS23* [21], *TERF1* [22], *TSRF1* [23], *PYL5* [24], *OsNAC10* [25], *OsDIL* [26], and *GUDK* [27] enhance drought tolerance by activating stress-responsive genes modulating ABA and JA signaling, boosting ROS-scavenging enzymes, or strengthening root-mediated water uptake to maintain growth under water deficit conditions. Epigenetic mechanisms, including H3K4-trimethylation [28] and circadian rhythm regulation via *OsPYL9* [29], along with ABA catabolism through *OsABA8ox3* [30], fine-tune gene expression to optimize drought responses. Conversely, miR164-targeted *NAC* genes [31], such as *OsFTIP6-OsHB22-OsMYBR57* [32], and inducible transcription factors from Arabidopsis and rice [33], orchestrate adaptive gene networks, while silicon enhances drought tolerance by upregulating *NACs* [34] and *DREB2A* [34], conferring drought tolerance in rice. Collectively, the coordinated action of functional genes, transcription factors, hormonal pathways, and epigenetic mechanisms plays a critical role in drought tolerance in rice, offering promising targets for improving drought stress tolerance and sustaining crop productivity under water-limited conditions.

FAD-linked oxidoreductase proteins are major plant flavoenzymes that bind FAD and mediate electron transfer in diverse metabolic and signaling pathways. These proteins are characterized by a well-defined FAD-binding domain that stabilizes the flavin cofactor within a Rossmann-like fold, enabling efficient electron transfer during redox homeostasis, modulating growth and adaptive response in plants [35]. Several studies have reported that FAD-linked oxidoreductase proteins perform important functions in plant growth and development. For instance, the brassinosteroid (BR) biosynthetic gene *Ihhd10*, which encodes a putative FAD-linked oxidoreductase in rice, influences plant development by modulating the expression of key flowering regulators such as *OsMADS50* and reducing chlorophyll biosynthesis in late heading and altered plant heights [36]. In rice, natural variation in the *BRD2* allele, which encodes a FAD-linked oxidoreductase, altered plant heights by affecting internode cell number and influencing grain size through changes in cell expansion during spikelet development [37]. The role of FAD-dependent oxidoreductase proteins in response to stress conditions has been evaluated in several plants. For example, in Arabidopsis, FAD-dependent oxidoreductase proteins participate not only in primary metabolism but also in processes involved in development and responses to biotic and abiotic stresses [38]. In cotton, GhBBE59, a FAD-linked oxidoreductase, enhances stress tolerance by regulating cellular redox balance and activating antioxidant pathways under cadmium stress conditions [39]. A previous study identified several FAD-like oxidoreductases within the genomic region associated with multiple salinity stress traits, indicating these genes may contribute to salt stress adaptation in plants [40]. In Arabidopsis, UV-B stress activates the kinase GCN2, which phosphorylates eIF2α and induces stress-responsive genes, including FAD-linked oxidoreductases [41]. In *Ralstonia solanacearum*, the FAD-linked oxidase RSc0454 is critical for pathogenicity, regulating redox balance and virulence gene expression [42]. Together, these findings highlight FAD-linked oxidoreductases as conserved regulators for plant growth, development, and stress adaptation.

Previous studies have demonstrated that FAD-linked oxidoreductase proteins participate in various biological processes, including hormone biosynthesis, growth regulation, and responses to biotic and abiotic stresses. In rice, the FAD-linked oxidoreductase proteins such as BRD2 and Ihhd10 are essential for plant height, heading date, and grain development [36,37], whereas studies in other species linked this protein family to redox regulation and stress adaptation. However, the role of FAD-linked oxidoreductase proteins in drought stress, as well as the molecular machinery behind the FAD-linked oxidoreductase proteins in grain development, remains largely unexplored. In the present study, we characterized a previously unreported FAD-linked oxidoreductase, *OsFLO1*, and demonstrated its dual role in regulating drought responses and grain development of rice, thereby providing new insights into the functional diversity of this family in crop plants.

## 2. Results

### 2.1. Identification of a Significant Locus Associated with Grain and Drought Tolerance Traits

**Figure 1:**
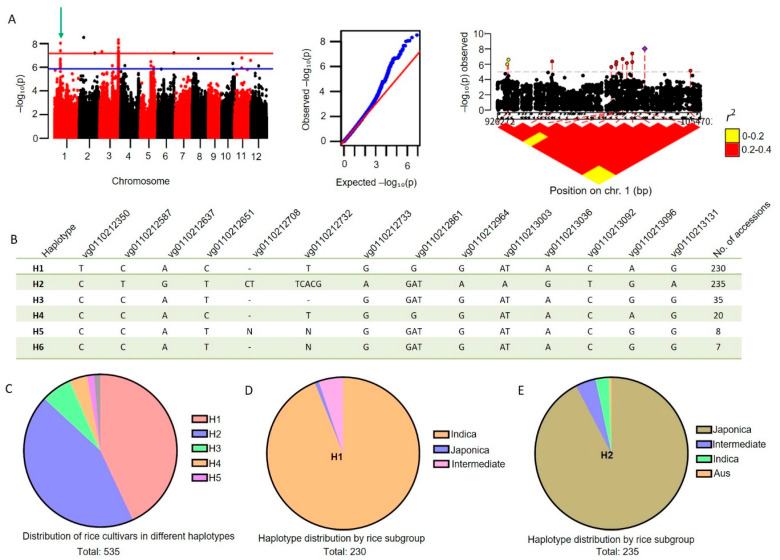
Genetic variation and haplotype analysis of a grain trait locus in rice. (**A**) Manhattan and quantile-quantile plots for GWAS of grain length on chromosome 1. The green arrow indicates the peak corresponding to the target gene in the GWAS data. (**B**) The haplotypes and their subspecies distribution of *OsFLO1* based on their representative variations in 535 accessions. Nucleotide variants are shown; dashes (-) indicate deletions and “N” denotes ambiguous calls. Numbers on the right represent haplotype frequencies in the studied population (n = 535). (**C**) Pie graph representing the distribution of rice cultivars across different haplotypes H1–H5. Each color represents a distinct haplotype: H1 (red), H2 (blue), H3 (green), H4 (Yellow), and H5 (purple). The gray represents accessions belonging to minor not included in H1–H5. (**D**,**E**) Subpopulation differentiation of major haplotypes H1 and H2 between *indica* and *japonica*.

Genome-wide association studies (GWAS) were conducted using a panel of 535 rice accessions with diverse origins to identify genetic loci associated with grain-related traits. On chromosome 1, GWAS identified a region with a prominent peak (>4) exceeding the genome-wide significance threshold (Figure 1A). The region was selected as a candidate for haplotype-based fine mapping and functional analysis. Six major haplotypes (H1–H6) were identified within this candidate region based on sequence polymorphisms, including SNPs and indels (Figure 1B). H1 and H2 were the most common haplotypes, detected in 230 and 235 accessions, respectively, whereas H3, H4, H5, and H6 were present at low frequencies (Figure 1B,C). The haplotype cluster analysis among the studied accessions suggested that *OsFLO1* could contribute to *indica*-*japonica* differentiation. For instance, H1 (*n* = 230) was present mainly in *Indica* cultivars, with a moderate frequency in Intermediate cultivars (Figure 1D). In contrast, H2 (n = 235) showed a strong preference for *Japonica*, with a moderate frequency in *Indica* and Intermediate cultivars (Figure 1E). Based on these results, we conclude that the distinct patterns of subpopulation distribution indicate that the haplotypes may have undergone selection during domestication and adaptation to different agroecological regions.

### 2.2. Geographical Distribution of OsFLO1 Haplotypes

**Figure 2:**
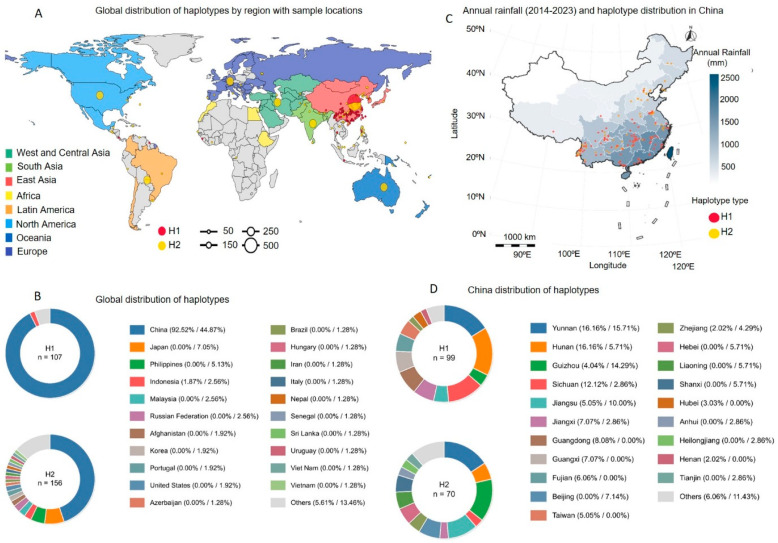
Global and China-specific distribution of haplotypes. (**A**) Global distribution of H1 (red) and H2 (yellow) with sample locations. Circle sizes represent sample number (small: 50, medium: 150, large: 250, extra-large: 500). Regions are color-coded as follows: West/Central Asia (dark green), South Asia (light green), East Asia (red), Africa (yellow), Latin America (orange), North America (light blue), Oceania (blue), and Europe (purple). (**B**) Global haplotype frequency distribution. Top: H1; Bottom: H2. Pie charts show the proportion of each haplotype in sampled countries, with percentages and counts indicated next to country names. (**C**) H1 (red) and H2 (yellow) distributions in China overlaid on average annual rainfall (2014–2023; darker shading = higher rainfall). (**D**) China-specific haplotype distribution. top: H1; bottom: H2. Pie charts show haplotype frequencies across provinces, with percentages indicated next to color-coded labels.

To better understand how the two major *OsFLO1* haplotype groups, including H1 and H2, associated with grain traits, derive their respective distributions, we plotted the geographic distribution of both haplotypes, H1 (red circles) and H2 (yellow circles), across the major rice-growing regions of the world (Figure 2). Overall, H1 is more widely distributed in Eastern Asia, as well as in parts of Latin America and Africa, whereas H2 is primarily concentrated in South Asia and Oceania, with a relatively high presence in both North and South America (Figure 2A). The diameter of each circle also represents the haplotype density in the corresponding geographical region. Further analysis was performed to examine haplotype frequencies by country, revealing distinct patterns of distribution for H1 and H2 across different regions, which may reflect historical selection pressures and adaptation to local agroecological conditions. Based on the data analysis of haplotype frequency, both haplotypes H1 and H2 were most prevalent in China, with frequencies of 87.6% and 42.6%, respectively (Figure 2B). Frequencies in countries not individually listed were generally low, with H1 and H2 accounting for 5.3% and 4.8%, respectively (Figure 2B). Similarly, unknown origins accounted for 5.3% and 4.8% for H1 and H2, and in Japan, H2 was present at 6.7% (Figure 2B). Overall, the geographic and population-specific prevalence of these haplotypes provides valuable insights into the evolutionary history and breeding selection of rice.

Findings from country-specific haplotype frequencies reveal a clear geographic structure corresponding to major rice subpopulations. In China, haplotype distributions were further analyzed in relation to decadal annual rainfall data from 2014 to 2023. The red dots representing H1 accessions were more abundant in the high-rainfall southern and southeastern provinces, whereas yellow dots representing H2 accessions were largely confined to the relatively drier northern and northeastern regions (Figure 2C). These observations suggest that local precipitation patterns may influence haplotype distribution, with H1 accession favoring regions with higher rainfall and H2 accession predominating in drier areas. This pattern highlights the potential role of environmental factors such as water availability in shaping the geographic prevalence of *OsFLO1* haplotypes, and may reflect historical adaptation and selection of rice varieties to specific agroecological conditions. Pie charts depicting haplotype preferences at the province level provided additional resolution of the geographical patterns described above, allowing a more detailed visualization of the distribution of H1 and H2 within individual provinces (Figure 2D). H1 made up at least 70% of accessions in the humid subtropical provinces of Hunan, Yunnan, Sichuan, Jiangxi, Guangdong, Guangxi, and Fujian, while H2 constituted over 60% of accessions in the provinces of Heilongjiang, Taiwan, and Jiangsu, as well as other regions where *Japonica* rice varieties are extensively grown under relatively drier conditions (Figure 2D). The presence of small-scale geographic patterns associated with *OsFLO1* haplotypes supports their potential role in the local adaptation of rice to diverse environmental gradients, particularly variation in water availability.

### 2.3. OsFLO1 Positively Regulates Grain-Related Traits and Yield Components

**Figure 3:**
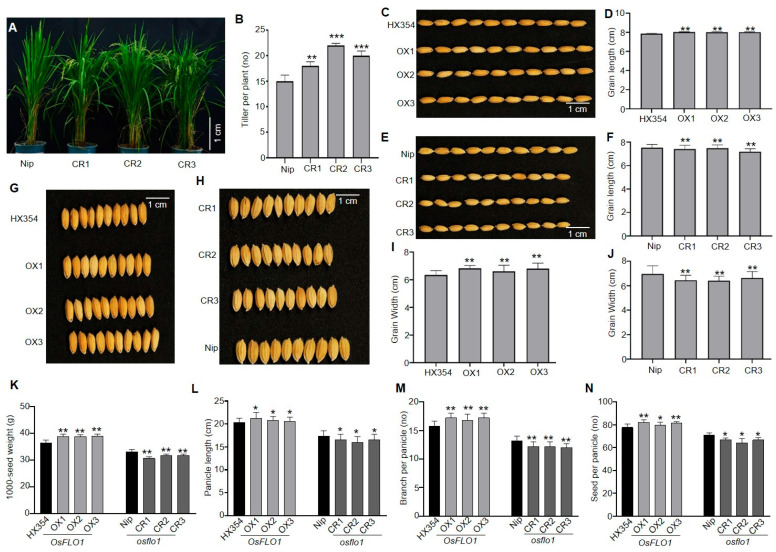
*OsFLO1* modulates grain-related traits in rice. Comparison of tiller number between WT (Nip) and *OsFLO1* knockout (CR) lines. (**A**) Representative plant images; (**B**) quantitative data. (**C**,**D**) Grain length of WT (HX354) and *OsFLO1* overexpression (OX) lines. (**C**) Representative grains; (**D**) quantitative measurements. (**E**,**F**) Grain length of *WT* (Nip) and *OsFLO1* knockout (CR) lines. (**E**) Representative grains; (**F**) quantitative measurements. (**G**,**I**) Grain width of *OsFLO1* overexpression (OX) lines and *WT*. (**G**) Representative grains: (**I**) quantitative measurements. (**H**,**J**) Grain length of WT and *OsFLO1* knockout (CR) lines. (**H**) Representative grains; (**J**) quantitative measurements. (**K**) 1000-seed weight. (**L**) Panicle length. (**M**) Branch number per panicle. (**N**) Seeds per panicle in WT, OX, and CR lines. Data are mean ± SD (n ≥ 10 plants). Asterisks indicate significant differences (* *p* < 0.05, ** *p* < 0.01, *** *p* < 0.001; Student’s *t*-test).

To investigate the effect of the *OsFLO1* gene on grain size development, we first evaluated the expression of *OsFLO1* in Nipponbare (Nip, a *japonica* cultivar) and HX353 (C418, an *indica* cultivar) in young panicles. *OsFLO1* showed higher expression in Nip, whereas lower expression was observed in HX353 (Figure S1). Therefore, we overexpressed *OsFLO1* in the HX353 background and generated a knockout by CRISPR/Cas9 of this gene in the Nip background. Three independent transgenic lines for overexpression (OX1, OX2, and OX3) and knockout (CR1, CR2, and CR3) lines were selected for further analysis based on the expression levels of the *OsFLO1* gene. Analysis of the T_3_ generation revealed that knockout of *OsFLO1* had a significant impact on tiller number, an important trait for yield. The *OsFLO1*-CR lines exhibited a higher number of tillers compared with the wild type (WT) (Figure 3A,B); however, no variation in tiller number was observed in the *OsFLO1*-OX lines, indicating that *OsFLO1* may be required for normal tiller development, but its overexpression does not further affect this trait. Next, we examined variation in grain size, including grain length and width. Our results indicate that *OsFLO1* potentially influences grain size. For instance, grain length increased upon overexpression of *OsFLO1* (Figure 3C,D), whereas it was reduced in the knockout lines (Figure 3E,F). Similarly, grain width was decreased in the *OsFLO1* knockout lines (Figure 3H,J) and increased in the overexpression lines (Figure 3G,I), suggesting that *OsFLO1* plays a significant role in determining grain size in rice. In addition to grain length and width, we evaluated 1000-seed weight, panicle length, number of branches per panicle, and number of seeds per panicle, as these traits are key determinants of rice yield and reproductive capacity. Consistent with the grain size results, overexpression of *OsFLO1* generally enhanced these yield-related traits, while knockout of the gene resulted in reductions. For instance, we observed that 1000-seed weight was increased in the *OsFLO1*-OX lines, where it was reduced in the knockout lines (Figure 3K). Similarly, panicle length was greater in the OX lines and shorter in the knockout lines (Figure 3L). The number of branches per panicle (Figure 3M) and the number of seeds per panicle (Figure 3N) also followed the same trend, with OX lines showing higher values and knockout lines showing a reduction compared with the WT. In conclusion, *OsFLO1* plays a positive regulatory role in multiple aspects of rice growth and yield. Overexpression of *OsFLO1* enhances grain size, 1000-seed weight, panicle length, number of branches per panicle, and seeds per panicle, while knockout of the gene reduces these traits. These results indicate that *OsFLO1* is a key determinant of both grain morphology and panicle architecture, making it a promising target for improving rice yield and grain quality through genetic manipulation in rice.

### 2.4. OsFLO1 Regulates Drought Tolerance in Rice

**Figure 4:**
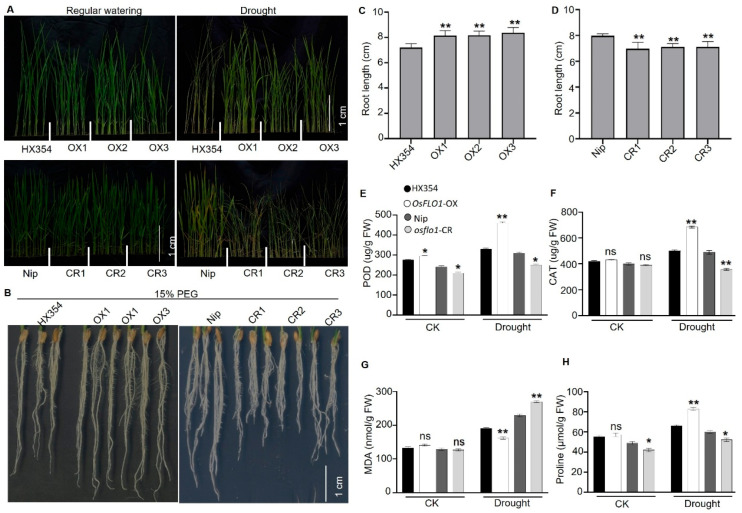
*OsFLO1* regulates growth and stress response under drought stress. (**A**) Drought performance of wild-type (HX354 and Nip), *OsFLO1* overexpression (OX), and knockout (CR) plants under drought stress. (**B**–**D**) Root growth of WT (HX354 and Nip), *OsFLO1* overexpression (OX), and knockout (CR) lines under 15% PEG-induced osmotic stress. (**B**) Representative root images; (**C**) root length of WT and OX lines; (**D**) root length of WT and CR lines. (**E**–**H**) Physiological and biochemical indicators in WT (HX354 and Nip), OX, and CR plants under control (CK) and drought conditions. (**E**) POD activity; (**F**) CAT activity; (**G**) MDA content; (**H**) proline content. Data are mean ± SD of three biological replicates (n ≥ 10 for each replicate). Statistical analysis compared with WT is indicated by asterisks: * *p* < 0.05, ** *p* < 0.01, ns: non-significant (Student’s *t*-test).

To explore the potential role of *OsFLO1* in drought stress, we evaluated the performance of the overexpression (OX) and knockout (CR) lines under water-limited conditions. Given the known involvement of FAD-lined oxidoreductase proteins in stress response, we hypothesize that *OsFLO1* might contribute to drought tolerance in addition to its role in grain development. By comparing physiological and morphological traits across the transgenic lines and WT, we aimed to determine whether *OsFLO1* influences rice response to water-deficient conditions. We performed a drought treatment by withholding water. The *OsFLO1* overexpression (OX) lines showed a higher survival rate compared with the wild type (HX354), whereas the knockout (CR) lines exhibited lower survival (Figure 4A). Root architecture is a key determinant of drought avoidance, as deeper and more extensive root systems enhance water uptake. Under 15% PEG-induced osmotic stress, root growth in *OsFLO1*-OX lines was significantly promoted, with longer primary and lateral roots (Figure 4B,C). In contrast, knockout lines developed shorter and less-branched root systems compared to the WT (Figure 4B,D). These results indicate that *OsFLO1* likely functions as a positive regulator of root elongation under stress conditions. These findings corroborate previous reports, highlighting the significant roles of *OsFLO1* in enhancing drought during vegetative growth. We evaluated the antioxidant responses by measuring the activities of catalase (CAT), peroxidase (POD), malondialdehyde (MDA), and proline content, as these parameters reflect the plant’s ability to cope with oxidative stress induced by drought. Under drought stress, peroxidase (POD) activity was reduced in the *OsFLO1* knockout lines, whereas it was increased in the overexpression (OX) lines compared with the WT (Figure 4E). A similar pattern was observed for catalase (CAT) activity, with increased levels in the *OsFLO1* overexpression (OX) lines and decreased levels in the knockout (CR) lines under drought stress (Figure 4F). However, a different pattern was observed for malondialdehyde (MDA), with lower levels in the *OsFLO1* overexpression (OX) lines and higher levels in the knockout (CR) lines under drought stress (Figure 4G). Moreover, the proline content increased in the *OsFLO1* overexpression (OX) lines and decreased in the knockout (CR) lines under drought stress (Figure 4H). In conclusion, *OsFLO1* enhances drought tolerance by modulating antioxidant and osmotic responses in rice. Overexpression of *OsFLO1* increased the activities of CAT and POD, elevated proline accumulation, and reduced MDA levels, indicating stronger ROS scavenging and reduced cellular damage under stress. Conversely, knockout of *OsFLO1* decreased antioxidant enzyme activities and proline contents while increasing MDA, reflecting weakened stress defense. These results suggest that *OsFLO1* plays a central role in protecting rice from oxidative and osmotic damage during drought conditions.

### 2.5. OsWRKY53 Specifically Binds to the OsFLO1 Promoter In Vitro and In Vivo

**Figure 5:**
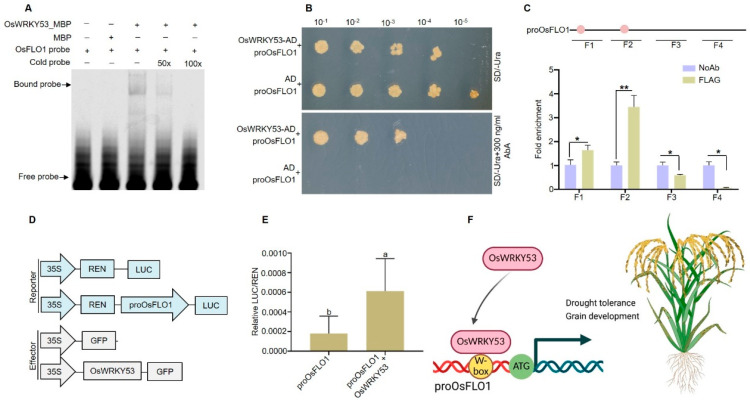
The binding of OsWRKY53 to the *OsFLO1* promoter regulates its function. (**A**) Electrophoretic mobility shift assay (EMSA) showing binding of OsWRKY53-MBP to a biotin-labeled *OsFLO1* promoter probe via W-box *cis*-element. (**B**) Y1H assay showing OsWRKY53 binding to the *OsFLO1* promoter. Growth on SD–Ura + AbA confirms the interaction. (**C**) ChIP-qPCR analysis of OsWRKY53 binding to the *OsFLO1* promoter. F1–F4 represent different promoter fragments; the fragments (F1 and F2) containing the W-box showed the highest enrichment compared to fragments lacking the W-box cis-element. Values are mean ± SEM (*n* = 3), the statistical significance was determined using a two-sided *t*-test, * *p* < 0.05, ** *p* < 0.01. (**D**) Construction of reporter and effector vectors for dual-luciferase transient expression (LUC/REN) analysis. (**E**) Dual-luciferase assay showing transcriptional activation of the *OsFLO1* promoter by OsWRKY53. Data are mean ± SD of three biological replicates. Bars annotated with different letters represent values that are significantly different (*p* ≤ 0.05) according to one-way ANOVA analysis. (**F**) The working mechanism of *OsFLO1* in rice involves regulation by OsWRKY53, which binds to the W-box in the *OsFLO1* promoter to modulate grain size and drought tolerance.

To further explore the regulatory mechanism underlying *OsFLO1* expression, we evaluated the binding of OsWRKY53 to the *OsFLO1* promoter. OsWRKY53 is a well-characterized transcription factor involved in multiple stress responses, including drought [43,44,45] and developmental processes such as grain [46,47,48,49] in rice. Given that *OsFLO1* positively regulates grain development, root architecture, and drought tolerance, we hypothesized that OsWRKY53 may directly modulate *OsFLO1* expression, linking transcriptional regulation to the physiological and stress-related phenotypes observed in the overexpression and knockout lines. We first performed an electrophoretic mobility shift assay (EMSA) to test the binding of OsWRKY53 to the *OsFLO1* promoter. EMSA revealed a band shift when OsWRKY53-MBP was incubated with a probe designed from the *OsFLO1* promoter containing the W-box (TGAC) *cis*-element, whereas this band shift was abolished by the addition of an unlabeled competitor probe (Figure 5A). This result confirms that OsWRKY53 directly binds to the W-box motif in the *OsFLO1* promoter, supporting its role as a transcriptional regulator of *OsFLO1*. We next performed a yeast one-hybrid (Y1H) assay to test the binding of OsWRKY53 to the *OsFLO1* promoter. Yeast cells co-transformed with *OsFLO1* promoter and OsWRKY53-AD were able to grow on SD-Ura medium containing 300 ng/µL AbA, whereas yeast containing *OsFLO1* promoter with the empty AD vector did not grow (Figure 5B). These results indicate that OsWRKY53 can specifically bind to the *OsFLO1* promoter in yeast. The interaction between OsWRKY53 and the *OsFLO1* promoter was further confirmed by the ChIP-qPCR, which showed that the promoter fragment containing the W-box *cis*-element exhibited higher fold enrichment compared with the region lacking the binding motif (Figure 5C). These results, together with those of Y1H and EMSAs, demonstrate that OsWRKY53 directly binds to the *OsFLO1* promoter in vivo and in vitro.

To determine whether OsWRKY53 acts as a transcriptional activator of *OsFLO1*, we performed a dual-luciferase assay in *Nicotiana benthamiana* leaves. To perform this assay, we first constructed the reporter and effector vectors (Figure 5D). The *OsFLO1* promoter drove firefly luciferase (LUC), while 35S-driven renilla luciferase (REN) served as an internal control. The constructed reporter and effector vectors were co-transformed into *Nicotiana benthamiana* leaves, and co-expression of *OsWRKY53*-*GFP* with the reporter containing the *OsFLO1* promoter increased LUC/REN activity three- to four-fold compared with the control containing only the reporter construct (Figure 5E), indicating that *OsWRKY53* may promote the expression of the *OsFLO1*. In conclusion, OsWRKY53 directly binds to the W-box *cis*-element in the *OsFLO1* promoter and acts as a transcriptional activator. This was supported by Y1H, EMSA, and ChIP-qPCR assays, as well as by dual-luciferase reporter analysis, which showed a three- to fourfold increase in LUC/REN activity upon co-expression with *OsWRKY53*. Together, these results demonstrate that OsWRKY53 positively regulates *OsFLO1* expression, providing a mechanistic link between transcriptional regulation and the observed effects of *OsFLO1* on grain development and drought tolerance.

## 3. Discussion

Rice is a staple crop for more than half of the world’s population, and grain yield and quality are key determinants of food security. Grain size, 1000-seed weight, and panicle architecture are major agronomic traits influencing rice productivity, while environmental stresses such as drought can severely limit grain development. Several studies, including genome-wide association studies (GWAS), have identified many genetic loci associated with grain size and drought tolerance in rice [50,51], but a few QTLs affect both traits, and their molecular mechanisms remain poorly understood. Although several genes regulating grain size and drought stress have been functionally characterized in rice, significant gaps remain in understanding how a single gene can coordinate both developmental and stress-responsive pathways. Here, our study aimed to fill this gap by evaluating the function of *OsFLO1* in regulating grain development and drought tolerance in rice, and we further discovered that OsWRKY53 acts as a transcriptional activator of *OsFLO1*, directly binding to its promoter and promoting its expression (Figure 5F). We believe that the regulatory module in which OsWRKY53 controls *OsFLO1* expression, linking transcriptional regulation to both grain development and drought tolerance, would provide a potential target for improving rice yield under stress conditions.

OsFLO1 belongs to the FAD-linked oxidoreductase family, a group of major plant flavoenzymes that regulate multiple functions, including growth and development and responses to biotic and abiotic stresses. For instance, in Arabidopsis, early leaf senescence in *cpr5/old* mutants has been associated with altered cellular redox balance, underscoring the importance of FAD-linked redox regulation in plant development and stress responses [52]. In rice, natural variation in the BRD2 allele has been shown to influence plant height and grain size, highlighting the role of FAD-linked oxidoreductase proteins in developmental trait regulation [37]. FAD-linked oxidase flavoenzymes have been shown to enhance stress resistance and metabolic regulation, enabling cotton to achieve cadmium-free harvest under stress conditions. Similarly, a recent study has reported the involvement of FAD-linked oxidoreductase protein in salt stress in plants [40]. Despite the involvement of FAD-linked oxidoreductases in diverse plant functions, their dual role in regulating both grain size and drought stress tolerance has not yet been reported. Here, in this study, the GWAS analysis identified the *OsFLO1* gene (Figure 1A), which regulates both grain size and drought tolerance in rice. Sequence polymorphisms, including SNPs and indels, divided the 535 accessions into six haplotypes (H1–H6) (Figure 1B). Most *indica* cultivars were grouped into H1, whereas *japonica* cultivars were predominantly assigned to H2 (Figure 1D,E). Similarly to our results, previous haplotype analysis has shown that major haplotypes can be strongly associated with *indica* vs. *japonica* groups in rice. For instance, chloroplast genome-based haplotype analysis revealed two dominant haplotypes largely corresponding to *japonica* and *indica* accessions, indicating subspecies-specific haplotype structuring [53]. Additionally, haplotype clustering of a grain size candidate gene (*OsLG3*) found distinct *indica* and *japonica* haplotype groups with contrasting geographic distribution, suggesting that differential haplotype patterns have arisen through domestication and selection [54].

So far, a few genes have been identified that regulate both of these important traits in rice. For instance, the orthologue of *OsSGL* has been reported to regulate drought tolerance as well as grain length and weight in rice [55]. A UDP-glucosyltransferase has been reported to regulate grain size while also enhancing tolerance to abiotic stresses in rice through metabolic flux redirection [56]. Similarly, another study reported that ectopic expression of the maize gene *ZmDUF1645* significantly increases grain length and yield, but compromises drought stress tolerance in rice [57]. Our study demonstrates that *OsFLO1* plays a central role in regulating grain development and drought tolerance in rice. For instance, overexpression of *OsFLO1* enhanced grain size (Figure 3B–J), 1000-seed weight (Figure 3K), and panicle traits (Figure 3L–N), while knockout lines exhibited reductions in these yield-related traits (Figure 3B–N). Under drought stress, *OsFLO1* overexpression improved root activity (Figure 4B–D), antioxidant enzyme activities (POD and CAT) (Figure 4E,F), proline accumulation (Figure 4H), and plant survival (Figure 4G), whereas knockout lines showed impaired stress responses and increased oxidative damage. While these results highlight the role of *OsFLO1* in enhancing antioxidant defenses, we cannot exclude the possibility that *OsFLO1* also influences other physiological or metabolic pathways that contribute to drought tolerance. Together, these results indicate that *OsFLO1* functions as a key regulator linking grain development and drought tolerance in rice, highlighting its potential as a target for breeding programs aimed at improving yield under water-limited conditions.

OsWRKY53, a well-characterized WRKY transcription factor, has been reported to regulate multiple aspects of plant growth and stress responses. For instance, several previous studies have shown that *OsWRKY53* contributes to disease resistance [44,58,59,60,61]. Like abiotic stresses, *OsWRKY53* is also critical for abiotic stress tolerance in rice [62,63,64]. Similarly, several studies have reported the contribution of *OsWRKY53* to the development of different traits in rice. For instance, *OsWRKY53* has been shown to positively regulate brassinosteroid signaling and plant architecture in rice, highlighting its broad role in developmental processes [65]. Previous research has shown that the OsWRKY53–OsGT1 regulatory module controls rice tiller development and fine-tunes strigolactone signaling, demonstrating that OsWRKY53 participates in diverse developmental processes [66]. *OsMKK70* regulates grain size and leaf angle in rice through the OsMKK4–OsMAPK6–OsWRKY53 signaling pathway, highlighting *OsWRKY53* as a key node linking MAPK signaling to developmental traits [48]. Our results demonstrate that OsWRKY53 acts as a transcriptional activator of *OsFLO1*, providing a mechanistic link between transcription regulation and the dual roles of *OsFLO1* in grain development and drought tolerance. In vivo and in vitro assays revealed that OsWRKY53 directly binds to the W-box *cis*-element in the *OsFLO1* promoter, promoting its role in regulating grain size and drought tolerance (Figure 5A–E). These findings position OsWRKY53 as a central regulatory hub, integrating upstream developmental and stress signals with the downstream activation of *OsFLO1* to coordinate grain size, panicle architecture, root growth, and drought tolerance in rice. We believe that the regulatory module in which OsWRKY53 controls *OsFLO1* expression, linking transcriptional regulation to both grain development and drought tolerance, would provide a potential target for improving rice yield under stress conditions.

## 4. Materials and Methods

### 4.1. Plant Materials and Growth Conditions

The rice cultivars *Nipponbare* (C146-*Nip*) and HX354 (C418-*Indica*) were used as wild-type (WT) plants during this study. All experiments were conducted at the National Key Laboratory of Crop Stress Resistance and High-Efficiency Production, College of Life Sciences, Northwest A&F University, Yangling, Shaanxi, China (34.31° N, 108.10° E, 435 m above sea level). Rice plants were grown in the experimental fields of Northwest A&F University during their normal growing season (late April to September). The average temperatures in Yangling during the growing season (late April to September) range from 25 °C to 35 °C. The space between plants within a row was 16.5 cm, and the space between rows was 26.5 cm. During this period, the fields were managed using standard agricultural practices, including irrigation, fertilization, and pest control, to ensure optimal rice growth.

### 4.2. Genome-Wide Association Study (GWAS)

The population structure of 535 accessions was used for GWAS as described previously [67]. The FaST-LMM method (Factored Spectrally Transformed Linear Mixed Model) was used to perform GWAS for both grain size and grain chalk [68]. The measured threshold for determining genome-wide significance for grain chalk was 1.37 × 10^−6^. Both Manhattan plots and LD block analysis were generated as described by Zhang et al., 2021 [69].

### 4.3. Sequence Variation and Haplotype Analysis

Sequence variation of *OsFLO1* from 533 rice accessions was obtained from the Rice Variation Map (RiceVarMap, http:/ricevarmap.ncpgr.cn) [70]. The 2 kb promoter region, the coding region, and the 0.5 kb downstream region were initially selected for the haplotype analysis. However, due to the large number of variations, we focused on the variation in the promoter region close to the start codon for haplotype analysis.

### 4.4. RNA Extraction and cDNA Synthesis for Cloning and Quantitative RT-PCR

Total RNA was extracted from samples collected from the Nip and HX354 background using TRIzol reagent (Invitrogen, Carlsbad, CA, USA) according to the manufacturer’s instructions. First-strand cDNA was synthesized with the PrimeScript RT reagent Kit (TaKaRa Bio, Shiga, Japan) following the manufacturer’s instructions. The qRT-PCR was performed on an RT-PCR system (QuantStudio 6 Flex, Applied Biosystems, Thermo Fisher Scientific, Waltham, MA, USA) using SYBR Green Master Mix (Roche, Basel, Switzerland). The *Ubiquitin* gene (LOC_Os03g13170) was used as the internal control. Relative expression levels were calculated using the 2^−ΔΔCt^ method. The primers used in this study are listed in the Supplementary Table S1.

### 4.5. Vector Construction and Plant Transformation

Since the expression of the *OsFLO1* gene was low in HX354 compared with Nip, we amplified the entire sequence (798 bp) of *OsFLO1* from Nip cDNA using KOD One^TM^ PCR Master Mix (TOYOBO, Osaka, Japan). The cloned fragment was digested with KpnI and BamHI enzymes (New England Biolabs, Ipswich, MA, USA) and subsequently cloned into the pCAMBIA1301-Flag vector using the ClonExpress II One Step Cloning Kit (Vazyme Biotech Co., Ltd., Nanjing, China; Cat. No. C112). The construct was driven by the maize *ubiquitin* (*Ubi*) promoter to construct *OsFLO1* overexpression lines. To generate *Osflo1* knockout mutants using the clustered regularly interspaced palindromic repeats/CRISPR-associated protein 9 (CRISPR/Cas9) system, guide RNA (gRNA) targeting the *OsFLO1* gene was designed and cloned into the pCXUN-Cas9 vector driven by the *OsU3* promoter [71]. All plasmids were verified using sequencing, and the constructs were introduced into *Agrobacterium tumefaciens* strain EHA105 for callus-based transformation. Hygromycin (50 mg/L) was used as the selective agent, and positive transgenic lines were identified by PCR and further confirmed by Sanger sequencing. The primers used in this study are listed in the Supplementary Table S1.

### 4.6. Morphological and Agronomic Trait Analysis

When the plant reached maturity, tiller number, panicle, and grain-related parameters were measured. To evaluate the drought tolerance of the plants, four-week-old rice seedlings were subjected to drought stress by withholding water for 21 days. Plants’ responses, such as leaf rolling, wilting, and survival rate, were monitored. To assess root activity, the rice seedlings were subjected to 10% polyethylene glycol (PEG-8000) solution to simulate drought conditions.

### 4.7. Measurement of Antioxidant Responses

Leaf tissues from WT (HX354 and Nip), *OsFLO1* overexpression (OX), and knockout (CR) rice plants were collected under the control and 15% PEG-induced osmotic stress. Samples were immediately frozen in liquid nitrogen and stored at −80 °C until analysis. Approximately 0.5 g of tissue was ground in 5 mL of ice-cold 50 mM Phosphate buffer (pH 7.0) containing 1% polyvinylpyrrolidone (PVP). The homogenate was centrifuged at 12,000× *g* for 20 min at 4 °C, and the supernatant was collected for enzyme assays. Enzyme activities (SOD, POD, and CAT) and metabolite contents (proline and MDA) were determined using commercial assay kits (Solarbio, Beijing, China) according to the manufacturer’s instructions.

### 4.8. Yeast One-Hybrid Assay

The yeast one-hybrid system was performed according to Matchmaker Gold Yeast One-Hybrid Library Screening System User Manual (Clontech, Mountain View, CA, USA). The promoter region (1 kb) of the *OsFLO1* gene was cloned into the *pAbAi* to construct the bait vector, and *OsWRKY53* was cloned into *pGADT7* to construct *OsWRKY53-AD*. The *pAbAi*-*OsFLO1* vector was introduced into yeast and grown on the synthetic medium lacking uracil (Ura). The minimal inhibitory concentration of *Aureobasidin* A (AbA) for the bait vector was determined on Ura-deficient medium. Subsequently, *pGADT7*-*OsWRKY53* was transformed into a yeast strain containing *pAbAi*-*OsFLO1* (bait) vector, and protein-DNA interactions were assessed on medium Ura-lacking medium containing 300 nm·ml^−1^ AbA.

### 4.9. Electrophoretic Mobility Shift Assay (EMSA)

The *OsWRKY53* CDS was cloned into *pMAL-c2X-MPB* to construct the *OsWRKY53-MBP* fusion vector, which was then purified in *Escherichia coli* BL21 (TransGen) using isopropyl β-D-1-thiogalactopyranosid (IPTG). The biotin-labeled and unlabeled competitor probes, approximately 24 bp, were designed from the DNA fragment containing the *cis*-element that provides the binding site for *OsWRKY53*. The DNA-binding reaction was incubated at room temperature (25 °C) for 30 min. The DNA-binding reaction was then electrophoresed using 6% acrylamide gel. After the electrophoresis, the DNA probes were transferred to a nylon membrane. The oligo bands were then detected using streptavidin-horseradish peroxidase (Beyotime, Shanghai, China) and the LightShift Chemiluminescent Electrophoretic Mobility Shift Assay (EMSA) kit (Thermo, Waltham, MA, USA). Competition assays were performed using unlabeled wild-type probes.

### 4.10. Chromatin Immunoprecipitation (ChIP) Assays

Chromatin immunoprecipitation was performed following the method described by Lee et al., 2017 [72]. The rice protoplast was treated with 1% formaldehydes to cross-link proteins and DNA, and the reaction was stopped by adding 2 M glycine. The chromatin was sonicated for 3–4 cycles (10 s On, 1 min OFF) and pre-cleared with 50 µL of protein G- Sepharose beads. Immunoprecipitation was then conducted using a 400-fold diluted monoclonal anti-GFP antibody. DNA was subsequently recovered using the phenol-chloroform extraction method. qRT-CPR was performed to assess the fold enrichment of *OsFLO1* promoter fragments in the OsWRKY53-GFP immunoprecipitates.

### 4.11. Dual-Luciferase Reporter Assay

The 1kb *OsFLO1* promoter region was cloned upstream of the *firefly luciferase* (*LUC*) gene in the pGreenII-0800-LUC vector. The coding sequence of *OsWRKY53* was cloned into 35S:pCAMBIA1301-GFP. Both constructs were co-transfected into *Nicotiana benthamiana* leaves via Agrobacterium-mediated infiltration. After 48 h, LUC and REN activities were measured using the Dual-Luciferase Reporter Assay System (Promega, Madison, WI, USA), and relative LUC/REN ratios were calculated from three biological replicates.

### 4.12. Statistical Analysis

All the experiments were performed with at least three biological replicates. Data are presented as mean ± standard deviation (SD). Statistical differences between groups were determined using Student’s *t*-test for two-group comparison or one-way ANOVA followed by Tukey’s multiple comparison test for multiple groups. Differences were considered statistically significant at *p* < 0.05, *p* < 0.01, and *p* < 0.001. For bar graphs with letters above bars, bars sharing the same letter are not significantly different. Graphs and statistical analyses were performed using GraphPad Prism v9.0.

## 5. Conclusions

In conclusion, *OsFLO1* plays a dual role by positively regulating grain development and enhancing drought tolerance. Overexpression of *OsFLO1* increased grain size, 1000-seed weight, panicle traits, root activity, antioxidant enzyme activities, and proline contents, while the knockout lines showed the opposite effect. We further demonstrated that OsWRKY53 acts as a transcriptional activator of *OsFLO1*, directly binding to its promoter and promoting its expression. Together, these findings reveal a molecular mechanism by which OsWRKY53-OsFLO1 coordinates yield-related traits and stress resilience, providing a potential target for breeding high-yield, drought-tolerant rice varieties.

## Data Availability

The data supporting this study can be found in the Supplementary Materials.

## Supplementary Materials

The following supporting information can be downloaded at: https://www.mdpi.com/article/10.3390/plants15071100/s1, Figure S1: Relative expression level of *OsFLO1* gene in HX354 and NIP plants; Table S1: List of primers used in this study.

## References

[1] Yang X., Wang B., Chen L., Li P., Cao C. (2019). The different influences of drought stress at the flowering stage on rice physiological traits, grain yield, and quality. Sci. Rep..

[2] Sadhukhan D., Mukherjee T., Sarkar A., Devi N.D., Bisarya D., Kumar V., Jincy M. (2024). A Comprehensive Analysis of Drought Stress Responses in Rice (*Oryza sativa* L.): Insights into Developmental Stage Variations from Germination to Grain Filling. Int. J. Environ. Clim. Change.

[3] Gupta A., Rico-Medina A., Caño-Delgado A.I. (2020). The physiology of plant responses to drought. Science.

[4] Liu C., Ma T., Yuan D., Zhou Y., Long Y., Li Z., Dong Z., Duan M., Yu D., Jing Y. (2022). The OsEIL1-OsERF115-target gene regulatory module controls grain size and weight in rice. Plant Biotechnol. J..

[5] Wang S., Li S., Liu Q., Wu K., Zhang J., Wang S., Wang Y., Chen X., Zhang Y., Gao C. (2015). The OsSPL16-GW7 regulatory module determines grain shape and simultaneously improves rice yield and grain quality. Nat. Genet..

[6] Hu J., Huang L., Chen G., Liu H., Zhang Y., Zhang R., Zhang S., Liu J., Hu Q., Hu F. (2021). The Elite Alleles of OsSPL4 Regulate Grain Size and Increase Grain Yield in Rice. Rice.

[7] Si L., Chen J., Huang X., Gong H., Luo J., Hou Q., Zhou T., Lu T., Zhu J., Shangguan Y. (2016). OsSPL13 controls grain size in cultivated rice. Nat. Genet..

[8] Yuan H., Qin P., Hu L., Zhan S., Wang S., Gao P., Li J., Jin M., Xu Z., Gao Q. (2019). OsSPL18 controls grain weight and grain number in rice. J. Genet. Genom..

[9] Fang N., Xu R., Huang L., Zhang B., Duan P., Li N., Luo Y., Li Y. (2016). SMALL GRAIN 11 Controls Grain Size, Grain Number and Grain Yield in Rice. Rice.

[10] Zhan P., Ma S., Xiao Z., Li F., Wei X., Lin S., Wang X., Ji Z., Fu Y., Pan J. (2022). Natural variations in grain length 10 (GL10) regulate rice grain size. J. Genet. Genom..

[11] Gao X., Zhang X., Lan H., Huang J., Wang J., Zhang H. (2015). The additive effects of GS3 and qGL3 on rice grain length regulation revealed by genetic and transcriptome comparisons. BMC Plant Biol..

[12] Wang Y., Lv Y., Yu H., Hu P., Wen Y., Wang J., Tan Y., Wu H., Zhu L., Wu K. (2023). GR5 acts in the G protein pathway to regulate grain size in rice. Plant Commun..

[13] Duan P., Ni S., Wang J., Zhang B., Xu R., Wang Y., Chen H., Zhu X., Li Y. (2015). Regulation of OsGRF4 by OsmiR396 controls grain size and yield in rice. Nat. Plants.

[14] Jia S., Xiong Y., Xiao P., Wang X., Yao J. (2019). OsNF-YC10, a seed preferentially expressed gene regulates grain width by affecting cell proliferation in rice. Plant Sci..

[15] She K.-C., Kusano H., Koizumi K., Yamakawa H., Hakata M., Imamura T., Fukuda M., Naito N., Tsurumaki Y., Yaeshima M. (2010). A Novel Factor *FLOURY ENDOSPERM2* Is Involved in Regulation of Rice Grain Size and Starch Quality. Plant Cell.

[16] Wang L., Wang D., Yang Z., Jiang S., Qu J., He W., Liu Z., Xing J., Ma Y., Lin Q. (2020). Roles of FERONIA-like receptor genes in regulating grain size and quality in rice. Sci. China Life Sci..

[17] Zhou Y., Miao J., Gu H., Peng X., Leburu M., Yuan F., Gu H., Gao Y., Tao Y., Zhu J. (2015). Natural Variations in *SLG7* Regulate Grain Shape in Rice. Genetics.

[18] Lyu J., Wang D., Duan P., Liu Y., Huang K., Zeng D., Zhang L., Dong G., Li Y., Xu R. (2020). Control of Grain Size and Weight by the GSK2-LARGE1/OML4 Pathway in Rice. Plant Cell.

[19] Yang X., Lu J., Shi W.-J., Chen Y.-H., Yu J.-W., Chen S.-H., Zhao D.-S., Huang L.-C., Fan X.-L., Zhang C.-Q. (2024). RGA1 regulates grain size, rice quality and seed germination in the small and round grain mutant srg5. BMC Plant Biol..

[20] Zhou L., Liu Z., Liu Y., Kong D., Li T., Yu S., Mei H., Xu X., Liu H., Chen L. (2016). A novel gene OsAHL1 improves both drought avoidance and drought tolerance in rice. Sci. Rep..

[21] Xu K., Chen S., Li T., Ma X., Liang X., Ding X., Liu H., Luo L. (2015). OsGRAS23, a rice GRAS transcription factor gene, is involved in drought stress response through regulating expression of stress-responsive genes. BMC Plant Biol..

[22] Gao S., Zhang H., Tian Y., Li F., Zhang Z., Lu X., Chen X., Huang R. (2008). Expression of TERF1 in rice regulates expression of stress-responsive genes and enhances tolerance to drought and high-salinity. Plant Cell Rep..

[23] Quan R., Hu S., Zhang Z., Zhang H., Zhang Z., Huang R. (2010). Overexpression of an ERF transcription factor *TSRF1* improves rice drought tolerance. Plant Biotechnol. J..

[24] Kim H., Lee K., Hwang H., Bhatnagar N., Kim D.-Y., Yoon I.S., Byun M.-O., Kim S.T., Jung K.-H., Kim B.-G. (2014). Overexpression of *PYL5* in rice enhances drought tolerance, inhibits growth, and modulates gene expression. J. Exp. Bot..

[25] Jeong J.S., Kim Y.S., Baek K.H., Jung H., Ha S.-H., Do Choi Y., Kim M., Reuzeau C., Kim J.-K. (2010). Root-Specific Expression of OsNAC10 Improves Drought Tolerance and Grain Yield in Rice under Field Drought Conditions. Plant Physiol..

[26] Guo C., Ge X., Ma H. (2013). The rice OsDIL gene plays a role in drought tolerance at vegetative and reproductive stages. Plant Mol. Biol..

[27] Ramegowda V., Basu S., Krishnan A., Pereira A. (2014). Rice GROWTH UNDER DROUGHT KINASE Is Required for Drought Tolerance and Grain Yield under Normal and Drought Stress Conditions. Plant Physiol..

[28] Zong W., Zhong X., You J., Xiong L. (2013). Genome-wide profiling of histone H_3_K_4_-tri-methylation and gene expression in rice under drought stress. Plant Mol. Biol..

[29] Usman B., Nawaz G., Zhao N., Liao S., Liu Y., Li R. (2020). Precise Editing of the *OsPYL9* Gene by RNA-Guided Cas9 Nuclease Confers Enhanced Drought Tolerance and Grain Yield in Rice (*Oryza sativa* L.) by Regulating Circadian Rhythm and Abiotic Stress Responsive Proteins. Int. J. Mol. Sci..

[30] Cai S., Jiang G., Ye N., Chu Z., Xu X., Zhang J., Zhu G. (2015). A Key ABA Catabolic Gene, OsABA8ox3, Is Involved in Drought Stress Resistance in Rice. PLoS ONE.

[31] Fang Y., Xie K., Xiong L. (2014). Conserved miR164-targeted NAC genes negatively regulate drought resistance in rice. J. Exp. Bot..

[32] Yang L., Chen Y., Xu L., Wang J., Qi H., Guo J., Zhang L., Shen J., Wang H., Zhang F. (2022). The OsFTIP6-OsHB22-OsMYBR57 module regulates drought response in rice. Mol. Plant.

[33] Datta K., Baisakh N., Ganguly M., Krishnan S., Shinozaki K.Y., Datta S.K. (2012). Overexpression of *Arabidopsis* and Rice stress genes’ inducible transcription factor confers drought and salinity tolerance to rice. Plant Biotechnol. J..

[34] Khattab H.I., Emam M.A., Emam M.M., Helal N.M., Mohamed M.R. (2014). Effect of selenium and silicon on transcription factors NAC5 and DREB2A involved in drought-responsive gene expression in rice. Biol. Plant..

[35] Dym O., Eisenberg D. (2001). Sequence-structure analysis of FAD-containing proteins. Protein Sci..

[36] Liu X., Feng Z.M., Zhou C.L., Ren Y.K., Mou C.L., Wu T., Yang C.Y., Liu S.J., Jiang L., Wan J.M. (2015). Brassinosteroid (BR) biosynthetic gene lhdd10 controls late heading and plant height in rice (*Oryza sativa* L.). Plant Cell Rep..

[37] Huang J., Chen Z., Lin J., Chen J., Wei M., Liu L., Yu F., Zhang Z., Chen F., Jiang L. (2022). Natural variation of the BRD_2_ allele affects plant height and grain size in rice. Planta.

[38] Schall P., Marutschke L., Grimm B. (2020). The Flavoproteome of the Model Plant *Arabidopsis thaliana*. Int. J. Mol. Sci..

[39] Malik W.A., Afzal M., Yousuf S., Ali M., Sahu S.K., Malook S.U. (2025). Oxidizing the odds: FAD-linked oxidase flavoenzymes empower cotton for cadmium-free harvests. Ind. Crop. Prod..

[40] Kang Y., Torres-Jerez I., An Z., Greve V., Huhman D., Krom N., Cui Y., Udvardi M. (2018). Genome-wide association analysis of salinity responsive traits in *Medicago truncatula*. Plant Cell Environ..

[41] Llabata P., Richter J., Faus I., Słomiňska-Durdasiak K., Zeh L.H., Gadea J., Hauser M.-T. (2019). Involvement of the eIF2α Kinase GCN2 in UV-B Responses. Front. Plant Sci..

[42] Hu X., Zhao Z., Zhuo T., Fan X., Zou H. (2019). The RSc0454-Encoded FAD-Linked Oxidase Is Indispensable for Pathogenicity in *Ralstonia solanacearum* GMI1000. Mol. Plant-Microbe Interact..

[43] Huang K., Wu T., Ma Z., Li Z., Chen H., Zhang M., Bian M., Bai H., Jiang W., Du X. (2021). Rice Transcription Factor OsWRKY55 Is Involved in the Drought Response and Regulation of Plant Growth. Int. J. Mol. Sci..

[44] Xie W., Li X., Wang S., Yuan M. (2022). OsWRKY53 Promotes Abscisic Acid Accumulation to Accelerate Leaf Senescence and Inhibit Seed Germination by Downregulating Abscisic Acid Catabolic Genes in Rice. Front. Plant Sci..

[45] Yu Y., Zhang L. (2022). Overexpression of TaWRKY53 enhances drought tolerance in transgenic Arabidopsis plants. S. Afr. J. Bot..

[46] Tian X., He M., Mei E., Zhang B., Tang J., Xu M., Liu J., Li X., Wang Z., Tang W. (2021). WRKY53 integrates classic brassinosteroid signaling and the mitogen-activated protein kinase pathway to regulate rice architecture and seed size. Plant Cell.

[47] Tang J., Mei E., He M., Bu Q., Tian X. (2022). Functions of OsWRKY24, OsWRKY70 and OsWRKY53 in regulating grain size in rice. Planta.

[48] Liu Z., Mei E., Tian X., He M., Tang J., Xu M., Liu J., Song L., Li X., Wang Z. (2021). OsMKKK70 regulates grain size and leaf angle in rice through the OsMKK4-OsMAPK6-OsWRKY53 signaling pathway. J. Integr. Plant Biol..

[49] Zhao H., Fu Y., Luo Y., Yang W., Gao Y., Liang X., Zhang Y., Zhao D., Li S., Li F. (2025). GL6.1 integrates grain length regulation and disease resistance via OsWRKY53-mediated signaling in rice. Crop J..

[50] Huang X., Zhao Y., Wei X., Li C., Wang A., Zhao Q., Li W., Guo Y., Deng L., Zhu C. (2011). Genome-wide association study of flowering time and grain yield traits in a worldwide collection of rice germplasm. Nat. Genet..

[51] Varshney R.K., Sinha P., Singh V.K., Kumar A., Zhang Q., Bennetzen J.L. (2020). 5Gs for crop genetic improvement. Curr. Opin. Plant Biol..

[52] Jing H., Hebeler R., Oeljeklaus S., Sitek B., Stühler K., Meyer H.E., Sturre M.J.G., Hille J., Warscheid B., Dijkwel P.P. (2008). Early leaf senescence is associated with an altered cellular redox balance in *Arabidopsis cpr5*/*old1* mutants. Plant Biol..

[53] Cheng L., Nam J., Chu S.-H., Rungnapa P., Min M.-H., Cao Y., Yoo J.-M., Kang J.-S., Kim K.-W., Park Y.-J. (2019). Signatures of differential selection in chloroplast genome between japonica and indica. Rice.

[54] Yu J., Xiong H., Zhu X., Zhang H., Li H., Miao J., Wang W., Tang Z., Zhang Z., Yao G. (2017). OsLG3 contributing to rice grain length and yield was mined by Ho-LAMap. BMC Biol..

[55] Liu K., Li M., Zhang B., Yin X., Xia X., Wang M., Cui Y. (2022). Poaceae Orthologs of Rice OsSGL, DUF1645 Domain-Containing Genes, Positively Regulate Drought Tolerance, Grain Length and Weight in Rice. Rice Sci..

[56] Dong N.-Q., Sun Y., Guo T., Shi C.-L., Zhang Y.-M., Kan Y., Xiang Y.-H., Zhang H., Yang Y.-B., Li Y.-C. (2020). UDP-glucosyltransferase regulates grain size and abiotic stress tolerance associated with metabolic flux redirection in rice. Nat. Commun..

[57] Li Y., Wang W., Hu C., Yang S., Ma C., Wu J., Wang Y., Xu Z., Li L., Huang Z. (2023). Ectopic Expression of a Maize Gene *ZmDUF1645* in Rice Increases Grain Length and Yield, but Reduces Drought Stress Tolerance. Int. J. Mol. Sci..

[58] Jimmy J.L., Babu S. (2015). Role of OsWRKY transcription factors in rice disease resistance. Trop. Plant Pathol..

[59] Chujo T., Miyamoto K., Ogawa S., Masuda Y., Shimizu T., Kishi-Kaboshi M., Takahashi A., Nishizawa Y., Minami E., Nojiri H. (2014). Overexpression of Phosphomimic Mutated OsWRKY53 Leads to Enhanced Blast Resistance in Rice. PLoS ONE.

[60] Xie W., Li X., Yue X., Zuo S., Yuan M. (2025). *OsVQ32*–*OsWRKY53* Module Regulates Rice Resistance to Bacterial Blight by Suppressing *OsPrx30*-Mediated ROS Scavenging. J. Agric. Food Chem..

[61] Hu L., Ye M., Li R., Lou Y. (2016). OsWRKY53, a versatile switch in regulating herbivore-induced defense responses in rice. Plant Signal. Behav..

[62] Li J., Chen Y., Zhang R., Wu B., Xiao G. (2023). Expression identification of three *OsWRKY* genes in response to abiotic stress and hormone treatments in rice. Plant Signal. Behav..

[63] Yu J., Zhu C., Xuan W., An H., Tian Y., Wang B., Chi W., Chen G., Ge Y., Li J. (2023). Genome-wide association studies identify OsWRKY53 as a key regulator of salt tolerance in rice. Nat. Commun..

[64] An S., Lü J., Ma Z., Gao X., Zhang B., Yang P., Ke Y. (2025). WRKY53: A Key Player in Stress Responses and Growth Regulation in Rice. Rice Sci..

[65] Tian X., Li X., Zhou W., Ren Y., Wang Z., Liu Z., Tang J., Tong H., Fang J., Bu Q. (2017). Transcription Factor OsWRKY53 Positively Regulates Brassinosteroid Signaling and Plant Architecture. Plant Physiol..

[66] Tang J., Zhao G., Yang J., Chen Z., Hong Z., Jin X., Qiu Z., Wang Z., Li X., Yan J. (2026). OsWRKY53-OsGT1 Module Regulates Rice Tiller Development and Is Involved in Fine-Tuning Strigolactone Signaling. Plant Biotechnol. J..

[67] Xie W., Wang G., Yuan M., Yao W., Lyu K., Zhao H., Yang M., Li P., Zhang X., Yuan J. (2015). Breeding signatures of rice improvement revealed by a genomic variation map from a large germplasm collection. Proc. Natl. Acad. Sci. USA.

[68] Lippert C., Listgarten J., Liu Y., Kadie C.M., Davidson R.I., Heckerman D. (2011). FaST linear mixed models for genome-wide association studies. Nat. Methods.

[69] Zhang J., Zhang D., Fan Y., Li C., Xu P., Li W., Sun Q., Huang X., Zhang C., Wu L. (2021). The identification of grain size genes by RapMap reveals directional selection during rice domestication. Nat. Commun..

[70] Zhao H., Yao W., Ouyang Y., Yang W., Wang G., Lian X., Xing Y., Chen L., Xie W. (2014). RiceVarMap: A comprehensive database of rice genomic variations. Nucleic Acids Res..

[71] He Y., Zhu M., Wang L., Wu J., Wang Q., Wang R., Zhao Y. (2018). Programmed Self-Elimination of the CRISPR/Cas9 Construct Greatly Accelerates the Isolation of Edited and Transgene-Free Rice Plants. Mol. Plant.

[72] Lee J.H., Jin S., Kim S.Y., Kim W., Ahn J.H. (2017). A fast, efficient chromatin immunoprecipitation method for studying protein-DNA binding in Arabidopsis mesophyll protoplasts. Plant Methods.

