# Supplementary material for: FAD-Linked Oxidoreductase Protein 1 (FLO1) Coordinates Grain Development and Drought Tolerance in Rice
Source: Plants (Basel). 2026 Apr 3;15(7):1100. doi: 10.3390/plants15071100 (PMC13074715; doi:10.3390/plants15071100)
Supplement: Supplementary file 1 [file plants-15-01100-s001.zip › plants-4099013-supplementary.pdf]

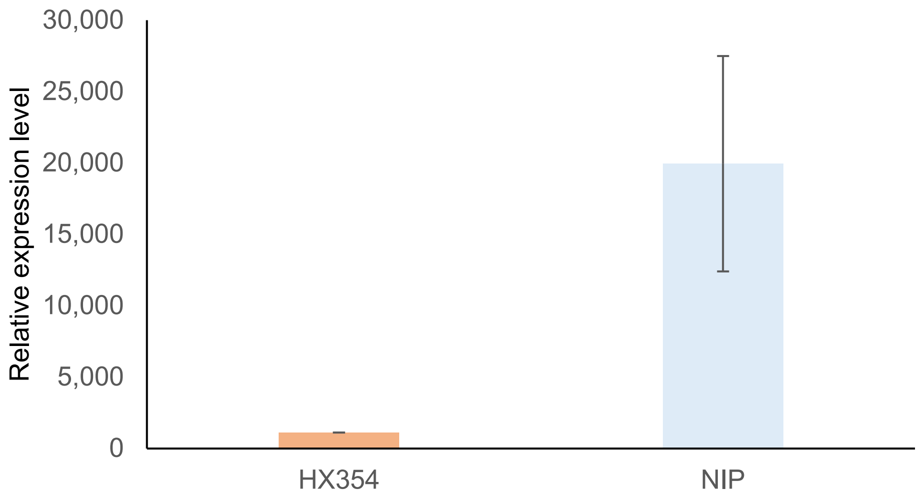


Figure S1: Relative Expression Level of OsFLO1 gene HX354 and NIP plants

Table S1. List of primers used in this study.

| Name | Forward primer | Reverse primer |
|---|---|---|
| OsFLO1-qRT | CTACGCTCATGACGAGACGG | TTAGCACTGTATGCCTCAGCA |
| OsFLO1-U3 | AGTCTACGCTCATGACGAGAgttttagagctagaaatagcaagtta | TCTCGTCATGAGCGTAGACTgccacggatcatctgcacaactc |
| OsFLO1-OX | TACGAACGATAGCCGGTACCATGGAGCCAAGCCGCTCG | TTGTAATCGGATCCGGTACCGCCTCTTCTTCCCCCTCT |
| OsFLO1-GFP | TCGAGCTTTCGCGAGCTCATGGAGCCAAGCCGCTCG | GCATGCCTGCAGGTCGACGCCTCTTCTTCCCCCTCT |
| proOsFLO1-AbAi | TGATGAATTGAAAAGCTTGCTTGGCTCCATGCTCGT | CAGAGCACATGCCTCGAGTACCAGTACTTTGGTACG |
| proOsFLO1-F1 | TCATGTGTTGCGGTTAGA | TACCAGTACTTTGGTACG |
| proOsFLO1-F2 | AGAGGGGTGAAGAAGAGG | ACACCGAAGGTCCCTCAAC |
| proOsFLO1-F3 | GCCAAGAGGAAGAGGACC | CTTCATCGCCCTCTTGGC |
| pro OsFLO1-F4 | GCTTGGCTCCATGCTCGT | CGCCGTTAAGTGAGGGAA |
| OsFLO1-probe | CGTATATGGATGACGTACCA | TGGTACGTCATCCATATACG |
| OsWRKY53-MBP | GAGGGAAGGATTTCAGAATTCATGGCGTCCTCGACGGGG | TGCCTGCAGGTCGACTCTAGACTAGCAGAGGAGCGACTC |
| OsWRKY53-Flag | GAATTCGAGCTCGGTACCATGGCGTCCTCGACGGGG | GTCGACTCTAGAGGATCCCTAGCAGAGGAGCGACTC |
| proOsFLO1-0800 | ggtcgacggtatcgataagcttGCTTGGCTCCATGCTCGT | ccgctctagaactagtggatccTACCAGTACTTTGGTACG |
